# Effects of collagen-induced rheumatoid arthritis on amyloidosis and microvascular pathology in APP/PS1 mice

**DOI:** 10.1186/1471-2202-12-106

**Published:** 2011-10-27

**Authors:** Sun Mi Park, Jin Hee Shin, Gyeong Joon Moon, Sung Ig Cho, Yong Beom Lee, Byoung Joo Gwag

**Affiliations:** 1 Department of Pharmacology, Ajou University School of Medicine, Suwon, South Korea; 2 Clinical research center, Samsung biomedical research institute, Seoul, South Korea; 3 GNT Pharma, Suwon, South Korea

## Abstract

**Background:**

Evidence suggests that rheumatoid arthritis (RA) may enhance or reduce the progression of Alzheimer's disease (AD). The present study was performed to directly explore the effects of collagen-induced rheumatoid arthritis (CIA) on amyloid plaque formation, microglial activation, and microvascular pathology in the cortex and hippocampus of the double transgenic APP/PS1 mouse model for AD. Wild-type or APP/PS1 mice that received type II collagen (CII) in complete Freund's adjuvant (CFA) at 2 months of age revealed characteristics of RA, such as joint swelling, synovitis, and cartilage and bone degradation 4 months later. Joint pathology was accompanied by sustained induction of IL-1β and TNF-α in plasma over 4 weeks after administration of CII in CFA.

**Results:**

CIA reduced levels of soluble and insoluble amyloid beta (Aβ) peptides and amyloid plaque formation in the cortex and hippocampus of APP/PS1 mice, which correlated with increased blood brain barrier disruption, Iba-1-positive microglia, and CD45-positive microglia/macrophages. In contrast, CIA reduced vessel density and length with features of microvascular pathology, including vascular segments, thinner vessels, and atrophic string vessels.

**Conclusions:**

The present findings suggest that RA may exert beneficial effects against Aβ burden and harmful effects on microvascular pathology in AD.

## Background

Alzheimer's disease (AD) is characterized by extracellular Aβ deposition, intracellular neurofibrillary tangles, neuronal loss, and cerebral microvascular pathology, resulting in progressive learning and memory deficits [1-3]. Extensive evidence supports a dynamic role for inflammation in the AD pathogenesis. Microglia, the macrophages that reside in the central nervous system, are activated around Aβ deposits in the brains of AD patients [4]. Levels of inflammatory mediators including TNF-α and C-reactive protein (CRP) are also increased in the peripheral blood of AD patients [5,6] and associated with increased risk of AD [7,8]. Activated microglia/macrophages derived from the brain and blood produce chemokines, cytokines, and free radicals, which participate in Aβ plaque formation and the neurodegenerative process in AD [9-13].

Several epidemiological studies have demonstrated that the relative risk of AD is significantly reduced in rheumatoid arthritis (RA) patients treated with nonsteroidal anti-inflammatory drugs (NSAIDs) for longer than 2 yrs [14,15]. In meta-analysis of 17 epidemiological studies, protective effects of arthritis and anti-inflammatory drugs have been observed against AD [15]. These studies have raised the hypothesis that anti-inflammatory drug treatment protects against AD, but arthritis or RA itself could modulate AD risk. Cyclooxygenase-2 (COX-2), an inducible cyclooxygenase known to mediate inflammation in RA and AD, has been proposed as a positive regulator for amyloid plaque formation and cognitive deficit in AD. In support of this hypothesis, administration of ibuprofen, a mixed COX-1/COX-2 inhibitor, reduces microglial activation, Aβ production, plaque burden, and cognitive impairment in Tg2576 mice [16,17]. Amyloid plaque formation is enhanced in APP/PS1 mice overexpressing human COX-2 [18]. However, the beneficial effects of NSAIDs have not been verified in randomized, double-blind, placebo-controlled clinical trials for AD patients [19]. In such clinical trials, NSAIDs were administered for less than 1 yr due to increased risk of acute myocardial infarction in patients receiving NSAIDs for longer than 18 months [20]. In a recent report from Alzheimer's disease anti-inflammatory prevention trial (ADAPT) with NSAIDs, treatment of asymptomatic individuals with naproxen for 2 yr was shown to reduce incidence of AD after 2 to 3 yr [21]. Therefore, the preventive and disease-modifying potential of NSAIDs remains to be determined in AD patients treated with NSAIDs for a long period of time (>2 yrs).

In a recent study, amyloid plaque formation and cognitive impairment were reduced in AD mice that were subjected to subcutaneous administration of granulocyte-macrophage colony-stimulating factor (GM-CSF), an inflammatory cytokine shown to increase in RA, for 20 d [22]. Combined with findings from epidemiological studies indicating that the incidence of AD is reduced in RA patients with prolonged NSAID treatment, RA is expected to directly modulate AD onset and progression. To examine this hypothesis, 2-month-old APP/PS1 mice were injected with bovine type II collagen (CII) in complete Freund's adjuvant (CFA), which has been widely used to induce RA in rodents [23]. Aβ burden, microglia/macrophages, and cerebral vascular pathology were investigated in APP/PS1 mice 4 months after the injections of CII in CFA to determine whether RA directly modulates amyloidosis and microvascular pathology in AD.

## Results

### Induction of collagen-induced arthritis (CIA) in APP/PS1 mice

**Figure 1:**
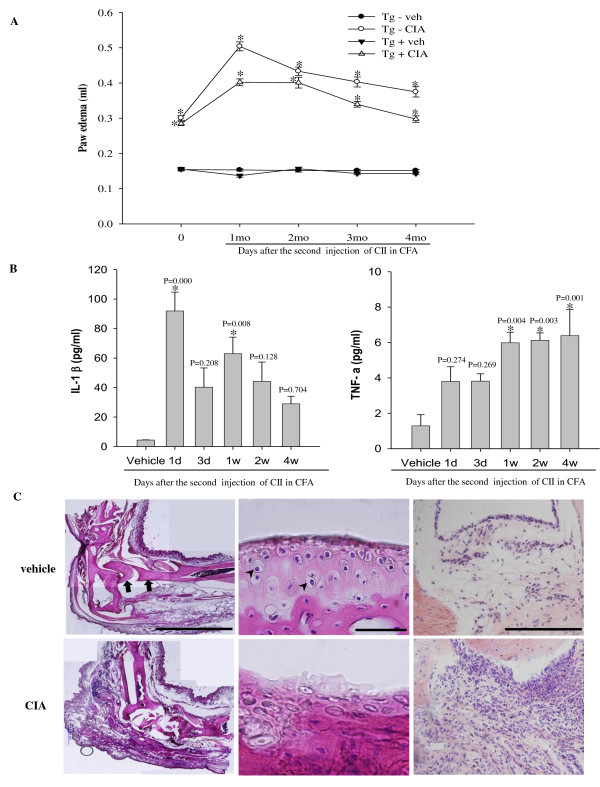
**Characterization of collagen-induced arthritis (CIA) in wild-type and APP/PS1 mice**. (**A**) Wild-type (Tg-) and APP/PS1 (Tg+) mice were subjected to injection with vehicle (Tg-vehicle, *n *= 16; Tg+ vehicle, *n *= 14), or with CII in CFA (Tg-CIA, *n *= 18; Tg+ CIA, *n *= 22) to induce CIA. Edema was observed at the right hind paw, which received the second injection of CII in CFA and was measured at indicated time points after injection. *, significant difference from relevant control. (**B**) ELISA of plasma IL-1β and TNF-α over 4 weeks after second injection with vehicle or CII in CFA in wild-type mice (*n *= 6 per group). (**C**) Bright-field photomicrographs showing ankle joints stained with hematoxylin and eosin in 6-month-old wild-type mice treated with vehicle (top row) or CIA (bottom row): left, low power of ankle joint (Arrows, bone; Scale bar = 2 mm); middle, high power of cartilage (Arrow heads, chondrocyte; Scale bar = 50 μm); right, high power of synovium (Scale bar = 200 μm). Note severe bone erosion, degradation of cartilage and chondrocytes, and intensive proliferation of synovial tissue in ankle joint 4 months after injection of CIA.

APP/PS1 mice and wild-type littermates injected with CII in CFA revealed pathologic features of RA. Paw swelling and redness of the joint were manifest immediately after the second immunization, reached a peak 1 month later, and significantly persisted over the next 4 months when animals were sacrificed (Figure 1A, P < 0.05). In contrast to CIA induced paw swelling in all four paws in DBA/1 mice [23], paw swelling was confined to the injection site in wild-type and APP/PS1 mice. Plasma levels of the pro-inflammatory cytokines IL-1β and TNF-α were increased within 1 d after the second immunization and remained elevated over the next 4 weeks in wild-type and APP/PS1 mice (Figure 1B, P *<*0.05). Paraffin-embedded joint sections stained with hematoxylin and eosin revealed severe bone erosion, cartilage/chondrocyte degradation, and proliferative synovitis in wild-type (Figure 1C) at 4 months after the second immunization. Such joint pathology was similar in APP/PS1 mice treated with CIA (data not shown).

### Effects of CIA on amyloid plaque pathology in APP/PS1 mice

**Figure 2:**
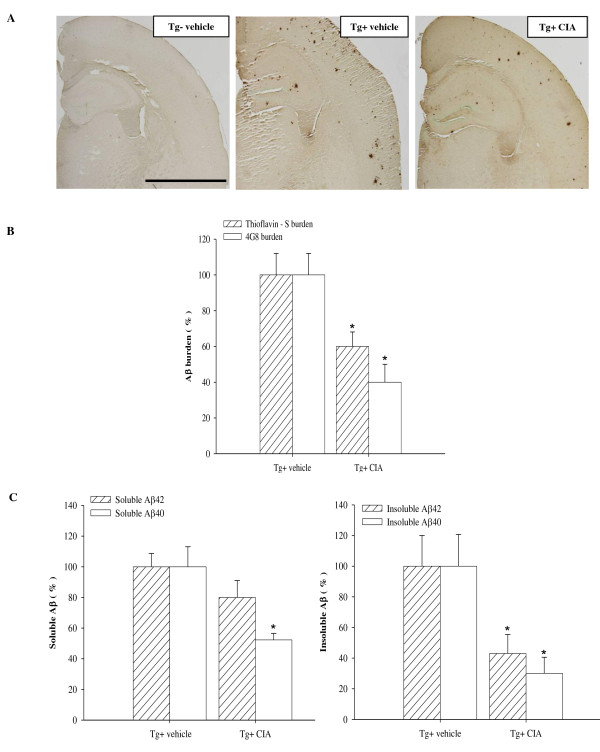
**CIA reduces amyloid plaque burden in APP/PS1 mice**. (**A**) Bright field photomicrographs of brain sections showing amyloid plaques immunolabeled with 4G8 in 6-month-old wild-type mice (Tg-vehicle) and APP/PS1 mice treated with vehicle (Tg+ vehicle) or CIA (Tg+ CIA). Scale bar = 2 mm. (**B**) Plaque burden was analyzed by measuring areas positive for 4G8 (Tg+ vehicle, *n *= 3; Tg+ CIA, *n *= 4) or thioflavine-S (Tg+ vehicle, *n *= 7; Tg+ CIA, *n *= 8). (**C**) ELISA of soluble and insoluble forms of Aβ40 and Aβ42 in combined cortical and hippocampal tissues (Tg+ vehicle, *n *= 7; Tg+ CIA, *n *= 8).

We examined the effect of CIA on Aβ deposition in APP/PS1 mice. APP/PS1 mice received CII in CFA at 2 months of age before Aβ deposition was evolved. When Aβ deposition was analyzed 4 months after CIA, levels of Aβ deposition were moderate in APP/PS1 mice [24]. Aβ plaques were widespread in the cerebral cortex and hippocampus of 6-month-old APP/PS1 mice (Figure 2A). Contrary to expectations, CIA substantially reduced Aβ plaque accumulation in APP/PS1 mice. Image analysis of Aβ deposition showed that Aβ plaques that were positive for 4G8 or thioflavine-S were significantly reduced in the cerebral cortex and hippocampus of 6-month-old APP/PS1 mice treated with CIA, compared with vehicle-treated APP/PS1 mice (Figure 2B). The effect of CIA on Aβ levels was analyzed in the opposite hemisphere using an ELISA that detects SDS-soluble and SDS-insoluble Aβ 40 and Aβ 42. Levels of soluble Aβ 40 and Aβ 42 appeared reduced by 48% (*P *= 0.056) and 20% (*P *= 0.026) in APP/PS1 mice 4 months after CIA, respectively, compared with vehicle-treated APP/PS1 mice. APP/PS1 mice treated with CIA revealed substantially significant reduction in levels of insoluble Aβ 40 and Aβ 42 (Figure 2C, P < 0.05).

### Enhanced activation of microglia/macrophage in APP/PS1 mice 4 months after CIA

**Figure 3:**
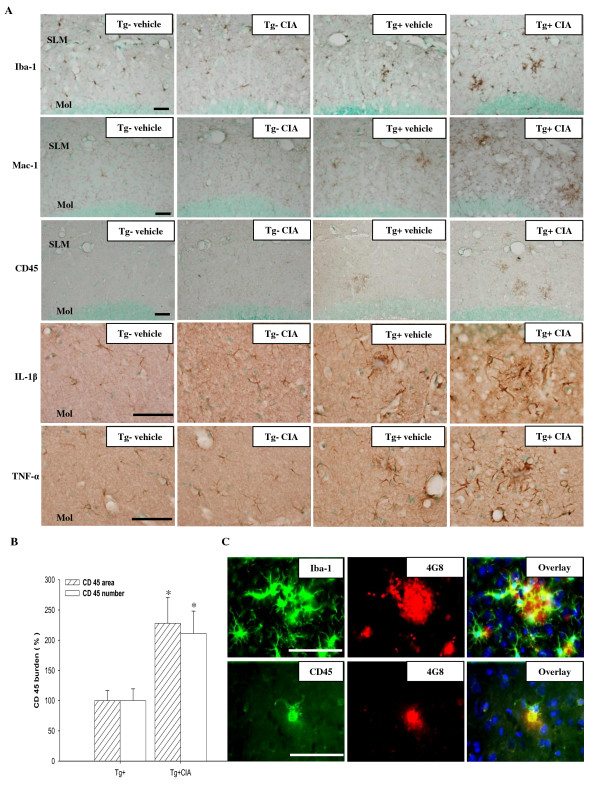
**CIA enhances activation of microglia/macrophages in APP/PS1 mice**. (**A**) Photomicrographs of the dentate gyrus molecular layer immunolabeled with antibodies against Iba-1, Mac-1, CD45, IL-1β, and TNF-α 4 months after injection of 2-month-old wild-type (Tg-) and APP/PS1 mice (Tg+) with vehicle or CIA. SLM, stratum lacunosum molecular; Mol, dentate gyrus molecular layer. Scale bar = 50 μm. (**B**) Activation of microglia/macrophages was analyzed by measuring the area and the number of CD45-immunoreactivity in the hippocampal and cortical regions (Tg+ vehicle, *n *= 3; Tg+ CIA, *n *= 4). (**C**) Fluorescence photomicrographs showing Iba-1 and CD45 signal around 4G8-positive amyloid plaques in the cortex of CIA-treated APP/PS1 mice. Top row, triple staining with Iba-1, 4G8, and DAPI (a nuclear marker); bottom row, triple staining with CD45, 4G8, and DAPI. Scale bar = 200 μm.

CIA-induced systemic inflammation likely triggers brain inflammation that contributes to internalization and degradation of Aβ through activated microglia. Iba-1 or Mac-1-immunoreactive microglial cells were observed in the brains of wild-type mice and increased in the brains of APP/PS1 mice at 6 months of age (Figure 3A). Immunoreactive Mac-1 positive cells were transiently increased in the cortex and hippocampus of wild-type mice over the course of 1 d after the second immunization injection. However, by 2 weeks after CIA Mac-1 immunoreactivity had returned to levels that were comparable to the levels in vehicle-injected mice (Additional file 1). In APP/PS1 mice treated with CIA, the number of Iba-1 or 1-immunoreactive cells in the cerebral cortex and hippocampus increased 4 months later. CD45-positive activated microglia or macrophages were not observed in wild-type mice at 4 months after CIA (Figure 3A). Activated microglia or macrophages were observed in the cortex of 6-month-old APP/PS1 mice and increased in the cerebral cortex and hippocampus of APP/PS1 mice treated with CIA up to approximately 210% (the number of CD45-immunoreactivity) and 230% (the area of CD45-immunoreactivity) (Figure 3A, B, *P *< 0.05). Immunoreactivity to IL-1β and TNF-α was increased in microglia and astrocytes in the molecular layer of the dentate gyrus of APP/PS1 mice compared to wild-type as previously reported [25-27]. CIA further increased expression of IL-1β and TNF-α in APP/PS1 mice (Figure 3A). In CIA-treated APP/PS1 mice, Iba-1-positive or CD45-positive cells were observed adjacent to Aβ plaques (Figure 3C).

### Effects of CIA on permeability of the blood-brain barrier (BBB) in APP/PS1 mice

**Figure 4:**
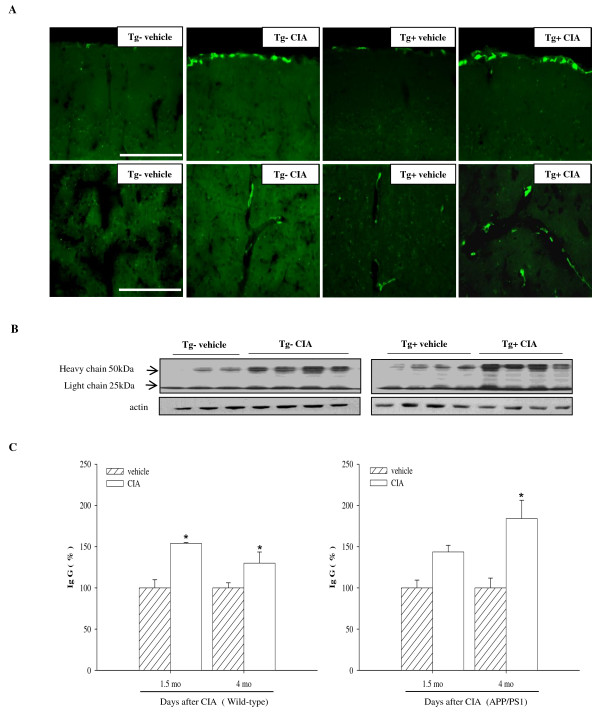
**CIA enhances macrophages at the leptomeningeal and perivascular regions and BBB permeability in APP/PS1 mice**. (**A**) Fluorescence photomicrographs of cortical areas immunolabeled with rat monoclonal anti-CD206 4 months after injection of 2-month-old wild-type (Tg-) and APP/PS1 (Tg+) mice with vehicle or CIA. Note increased leptomeningeal (top row) and perivascular (bottom row) macrophages in Tg- and Tg+ mice treated with CIA. Scale bar = 200 μm. (**B**) Western blot of IgG in combined cortical and hippocampal samples 4 months after CIA injection into Tg- and Tg+ mice. (**C**) IgG levels were analyzed 1.5 months (Tg- vehicle, *n *= 3; Tg- CIA, *n *= 3; Tg+ vehicle, *n *= 3; and Tg+ CIA, *n *= 4) and 4 months after vehicle or CIA injection into Tg- and Tg+ mice (Tg- vehicle, *n *= 3; Tg- CIA, *n *= 4; Tg+ vehicle, *n *= 4; and Tg+ CIA, *n *= 4).

CIA has been shown to increase BBB permeability in DBA/1 mice [28]. CIA likely causes the infiltration of monocytes and activation of microglia/macrophages that contribute to clearance of Aβ peptide in APP/PS1 mice [29]. Immunoreactivity to a macrophage mannose receptor (CD206) antibody was observed sparsely in the perivascular and leptomeningeal regions of wild-type and APP/PS1 mice. CD206-positive macrophages were increased in the vicinity of vessels in wild-type and APP/PS1 mice subjected to CIA (Figure 4A), suggesting the possibility that CIA induces the migration of macrophages into the brain parenchyma through the disrupted BBB. In support of this hypothesis, levels of immunoglobulin G (IgG) were significantly increased in the brains of wild-type and APP/PS1 mice 1.5 and 4 months after CIA compared with relevant vehicle (Figure 4B and 4C).

### CIA induces cerebral microvascular pathology in APP/PS1 mice

**Figure 5:**
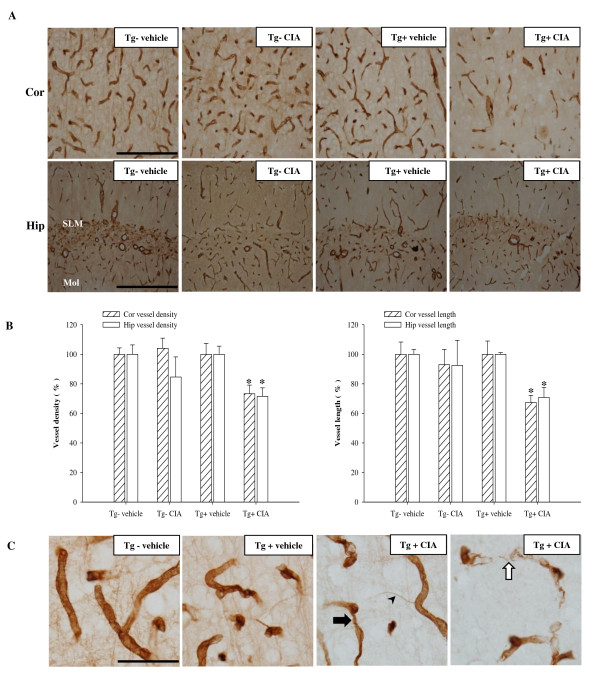
**CIA enhances microvascular pathology in APP/PS1 mice**. (**A**) Bright-field photomicrographs showing microvascular pathology in the cerebral cortex (Cor, top row) and hippocampus (Hip, bottom row) immunolabeled with collagen-IV 4 months after treatment of 2-month-old wild-type (Tg-) and APP/PS1 (Tg+) mice with vehicle or CIA. Scale bar = 200 μm. (**B**) Analysis of vessel density and length in the cerebral cortex and hippocampus (Tg- vehicle, *n *= 3; Tg- CIA, *n *= 3; Tg+ vehicle, *n *= 3; and Tg+ CIA, *n *= 4). (**C**) High-power photomicrographs showing microvascular pathology in the cortex of CIA-treated Tg+ mice. Note thinner vessel (arrow), string vessel (arrowhead), and degenerating vascular segments (open arrow) in Tg+ CIA compared with vehicle. Scale bar = 50 μm.

Finally, we examined whether CIA would cause cerebrovascular pathology. Cerebral microvessels were immunolabeled with an anti-collagen type IV antibody, a marker of the endothelial basement membrane. CIA significantly reduced vascular density and length in the cerebral cortex and hippocampus of APP/PS1 mice 4 months later (Figure 5, A and 5B). CIA also induced cerebral microvascular pathology, such as vascular segments and atrophic string vessels (Figure 5C). At 4 months after the injection procedure, survival of vehicle-treated APP/PS1 mice was approximately 93.3%, while the survival of CIA-treated mice was reduced to 76.9% (Additional file 2).

## Discussion

Administration of CII in CFA produces features of RA including joint swelling, bone and cartilage degeneration, synovitis, and infiltration of inflammatory cells at the injection sites accompanied by sustained elevation of IL-1β and TNF-1β in plasma of wild-type and APP/PS1 mice. CIA reduces levels of Aβ and plaque burden in the cortex and hippocampus of APP/PS1 mice. The reduced amyloidosis is associated with BBB disruption and activation of microglia/macrophage. In contrast to the clearance of Aβ plaques, CIA enhances microcerebral pathology and mortality in APP/PS1 mice.

Inflammation can reduce amyloidosis and cognitive deficit in transgenic mouse models of AD. Amyloid plaque formation is enhanced in APP/PS1 mice overexpressing human COX-2, the inducible isoform of cyclooxygenase that is essential for prostaglandin E2 (PGE_2_)-mediated inflammation [30]. Chronic administration of ibuprofen, a mixed COX-1/COX-2 inhibitor, prevents microglial activation, and reduces levels of inflammatory cytokines, Aβ, and amyloid plaque formation in Tg2576 mice [16,17,31]. The administration of COX-2 inhibitors prevents Aβ-mediated suppression of long-term plasticity and improves synaptic plasticity in Tg2576 [18]. COX-2-mediated PGE_2 _production can enhance amyloidosis by reducing the phagocytic function of microglia and stimulating Aβ production through activation of the prostaglandin E2 and E4 receptors [32,33].

Several lines of evidence suggest that inflammation negatively regulates amyloidosis and cognitive deficit in transgenic mouse models of AD. Overexpression of TGF-β1 or IL-6 reduces amyloid plaque formation through microglial activation in Tg2576 and TgCRND8 mice [34,35]. Administration of G-CSF or M-CSF induces brain inflammation and ameliorates amyloid pathology and cognitive deficit in transgenic AD mice, including Tg 2576 [36,37]. The intracerebral infection of Borna disease virus, which induces microglial cell activation, also reduces amyloid plaque formation in Tg2576 mice [38]. Furthermore, overexpression of soluble complement receptor-related protein y, a complement inhibitor, reduces microglial activation, produces neurodegeneration, and enhances Aβ production and accumulation in human amyloid precursor protein transgenic mice bearing the amyloidogenic K670M, N671L, and V717F mutations [39].

Aβ produced in the brains of APP/PS1 mice or AD patients induces the expression and secretion of chemotactic cytokines, such as monocyte chemotactic protein 1, which enhances the transmigration and differentiation of monocytes from the bloodstream [40-42]. Circulating monocytes are increased in CIA-treated mice or RA patients, and are expected to transmigrate through the BBB into the brain areas exposed to Aβ. The brain monocytes can be differentiated into macrophages by cytokines such as M-CSF and IL-6, which are induced by Aβ or after CIA [35,43-47]. Macrophages and activated microglia contribute to Aβ clearance. Alternatively, CIA may reduce the Aβ plaque burden by increasing passive transport to the periphery through increased BBB disruption.

Aβ accumulation in the cerebral vessel triggers BBB disruption by reducing the expression of tight junction proteins such as occludin, claudin-5, and zona occludins-1[48]. Aβ can induce oxidative stress, mitochondrial dysfunction, and the activation of caspase-dependent apoptotic pathways, possibly through mechanisms involving interaction with RAGE, resulting in endothelial cell degeneration [48-50]. In the present study, we provide evidence that CIA-treated APP/PS1 mice develop vascular segments and atrophic string vessels characteristic of cerebrovascular pathology observed in AD patients.

Alzheimer's disease is occasionally accompanied by epileptic seizures [51]. Cortical and hippocampal seizures are detected in three different APP transgenic mice models of AD [52]. Seizures are frequently observed in APP/PS1 mice at 3 and 4.5 months of age and correlated with mortality that peaks at 3 to 4 months of age [53]. In the present study, two APP/PS1 mice died at 3 to 4 months of age (6.7% mortality). In thirty nine APP/PS1 mice treated with CIA, nine mice died at 3 to 4 months of age (23.1% mortality). Thereafter, no death was observed. Thus, CIA may increase mortality of APP/PS1 mice by enhancing seizures, which is likely related with sustained inflammation and enhanced cerebrovascular pathology.

While further study is needed to delineate mechanisms underlying the effects of CIA on vascular pathology, elevated monocytes and neutrophils in CIA-treated APP/PS1 mice likely migrate to the cerebral vessel, release cytotoxic molecules such as superoxide, matrix metalloproteases, and TNF-α, and then contribute to endothelial cell degeneration and mortality in a cooperative way with Aβ. Recently, systemic administration of lipopolysaccharide for 6 weeks was shown to induce sustained microglial activation and tau hyperphosphorylation in the hippocampus of 3X Tg-AD mice harboring three mutant human genes (APP_K670N;M671L_, PS1_M146V_, and tau_P301L_) [54]. Considering that lipopolysaccharide triggers systemic inflammation and BBB disruption, CIA may exacerbate tau pathology as well as vascular pathology in AD.

## Conclusions

CIA induced before the onset of amyloid pathology reduces Aβ burden over 4 months in APP/PS1 mice, presumably by enhancing the clearance and efflux of Aβ. However, such beneficial effects of CIA are accompanied by enhanced microvascular pathology and mortality. The present findings suggest that transient systemic inflammation may be beneficial in lowering Aβ burden; however, the chronic systemic inflammation demonstrated in RA is a serious risk factor for cerebral microvascular pathology and hypoperfusion in AD, which likely lead to neurodegeneration and cognitive deficit.

## Methods

### Animal Preparation

All experiments were performed in accordance with the Guideline for Animal Experiments of Ajou University and GNT Pharma. APP/PS1 mice (APPswe/PS1dE9) were purchased from the Jackson Laboratory (Bar Harbor, ME, USA). These mice were generated by co-injection of chimeric APPswe (K595N, M596L) and an exon-9-deleted PS1 variant (dE9) vector controlled by a mouse prion promoter [55]. Male APP/PS1 transgenic mice were cross-bred with B6C3F1/J background females. APP/PS1 mice (Tg+) and wild-type littermates (Tg-) were determined by PCR analysis of tail DNA.

### Induction of CIA

Bovine CII (Chondrex, Redmond, WA, USA) was dissolved in 0.1 M acetic acid at a 4 mg/ml by stirring overnight at 4°C, added to an equal volume of CFA (Chondrex), and homogenized as described previously [23]. To induce CIA, 2-month-old female Tg+ and Tg-mice were injected intradermally at the tail base with 0.1 ml CII in CFA or 0.1 ml mineral oil as a vehicle. The animals received another injection of CII in CFA or mineral oil at the right hind paw 14 d after the primary immunization. Paw edema was measured using a plethysmometer (Ugo Basile, Comerio, Italy) monthly after the second immunization. After 4 months, 6-month-old mice were sacrificed and examined for AD pathogenesis on the basis of amyloid plaques and cerebrovascular pathology.

### Analysis of plasma IL-1β and TNF-α

Plasma cytokine levels were analyzed at various time points after the secondary injections of CII in CFA. Blood was collected via cardiac puncture and then transferred into a heparin-coated tube (Becton Dickinson, USA). The tube was placed on ice and centrifuged at 5000 rpm (1500 *g*) for 3 min. The supernatant was collected and stored at -80°C. Plasma levels of IL-1β and TNF-α were measured with a commercial ELISA kit (Biosource, Camarillo, CA, USA) according to the manufacturer's instructions.

### Tissue preparation

Animals were anesthetized with an intraperitoneal injection of chloral hydrate (400 mg/kg) and transcardially perfused with 0.9% NaCl. The right hind paw injected with CII in CFA was removed, fixed in 10% formalin solution at 4°C for 24 h, and decalcified in 10% EDTA solution at 4°C for 3 weeks. The tissues were then embedded in paraffin and cut into 8-μm-thick sections. The right brain hemisphere was immersion-fixed with 4% paraformaldehyde for 24 h, cryoprotected in 30% sucrose for 48 h, and cut into 18-μm-thick coronal cryosections. The hippocampal formation and cortex were dissected out from the left hemisphere and frozen at -80°C for protein assay.

### Aβ ELISA

Combined cortical and hippocampal tissues were homogenized in 0.5 ml Tris-buffered saline (TBS) containing protease inhibitor cocktail (Calbiochem, San Diego, CA, USA) and centrifuged at 14,000 rpm for 30 min at 4°C. The supernatant was collected and stored at -80°C for Western blot analysis of IgG. The pellet was sonicated in 0.5 ml of 2% SDS in TBS and centrifuged at 14,000 rpm for 40 min at 4°C. The supernatant was used for analysis of soluble Aβ. For analysis of insoluble Aβ, the pellet was sonicated with 0.5 ml 70% formic acid in water and then neutralized by 1:40 dilution into 1 M Tris-phosphate buffer (pH 11.0). Levels of Aβ 40 and Aβ 42 were analyzed using an ELISA kit (Biosource, Camarillo, CA, USA) according to the manufacturer's instructions.

### Immunohistochemistry

Brain sections were washed in phosphate-buffered saline, incubated in washed in 0.3% H_2_O_2 _and 0.25% Triton X-100 for 10 min at room temperature, and reacted with 10% horse serum for 1 h. Sections were then reacted overnight at 4°C with one of the following primary antibodies: mouse monoclonal anti-4G8 (1:1000, Signet), rat monoclonal anti-CD45 (1:500, Serotec), rabbit polyclonal anti-Iba-1 (1:1000, WAKO), rat monoclonal anti-Mac-1 (1:500, Serotec), goat polyclonal anti-IL-1β (1:200, R&D), goat anti-polyclonal anti-TNF-α (1:200, R&D), rat monoclonal anti-CD206 (1:500, Serotec), and rabbit polyclonal anti-collagen type IV (1:500, Millipore). For 4G8 immunostaining, sections were pretreated with 70% formic acid for 10 min to expose the Aβ epitope. The sections were reacted with anti-mouse, anti-rat, or anti-rabbit IgG fluorescent or biotin-conjugated (Vector, Burlingame, CA, USA) secondary antibody for 2 h. The biotin-labeled sections were incubated with avidin-biotin-peroxidase complex (ABC Elite kit, Vector) for 1 h and then visualized using 3,3'-diaminobenzidine tetrahydrochloride dehydrate (DAB, Vector).

### Image analysis of amyloid plaque burden, activated microglia/macrophages, and vascular pathology

Brain sections containing the hippocampal formation and cortex were immunolabeled with an antibody to 4G8 (amyloid plaque burden), CD45 (activated microglia/macrophage), or collagen type IV (vascular pathology). To identify amyloid plaques using thioflavine-S staining, the brain sections were incubated in 1% aqueous thioflavine-S (Sigma) for 10 min, followed by dehydration with 70% ethyl alcohol, and coverslipped with mounting medium. Three sections on the anatomically comparable plane for each animal were analyzed using MetaMorph 6.1 software (Universal Imaging Corp, Downingtown, PA, USA). The immunostained sections were captured using an Olympus BX 51 microscope (Olympus, Japan) at a magnification of 200×. The areas positive for 4G8, thioflavine-S, or CD45 in the hippocampal formation and cortex were subtracted from nonspecific signal from the same section and scaled to the vehicle group (= 100). The vascular pathology was analyzed in the area including the CA1 pyramidal layer and cortex by measuring vessel density and length immunolabeled with collagen type IV.

### Western blotting

BBB permeability was investigated by monitoring levels of IgG. Combined cortical and hippocampal samples (see above) were electrophoresed on an 8-10% SDS polyacrylamide gel and transferred to a nitrocellulose membrane. The membrane was pre-incubated with 5% nonfat dry milk and reacted with a biotinylated anti-mouse lgG (1:500, Vector) overnight at 4°C. The membrane was then reacted with avidin-biotin-peroxidase complex (ABC Elite kit, Vector) for 2 h and then with enhanced chemiluminescence reagents (Amersham, Buckinghamshire, UK) on X-ray film. Band intensity was quantified using Image Gauge 3.12 (Fuji PhotoFilm Co). The blot was stripped and probed with rabbit polyclonal anti-actin (1:1000, Sigma) to normalize the protein load among samples.

### Statistical analysis

All values are expressed as mean ± S.E.M. An independent-samples *t*-test was used to compare two samples. Analysis of variance (ANOVA) and the Student-Newman-Keuls test were used for multiple comparisons. All analyses were calculated using SPSS version 12.0 for Windows. Statistical significance was assumed at *P *< 0.05.

## Authors' contributions

SMP performed most experiments and JHS, GJM, SIC, and YBL participated in the discussion of data. BJG carried out the design of the study, interpretation of the data and preparation of the manuscript. All authors read and approved the final manuscript.

## Supplementary Material

Additional file 1 **Figure S1 - Transient increase of microglial cell proliferation in wild-type mice treated with CIA**. Photomicrographs of the dentate gyrus molecular layer immunolabeled with Mac-1 after second injection of CII in CFA in 2-month-old wild-type mice. The number of Mac-1-immunoreactive microglial cells was increased within 1 d after second injection of CII in CFA. This increase remained for over 1 wk, but disappeared within 2 wks (*n *= 4 per group). Scale bar = 200 μm. Click here for file

Additional file 2 **Figure S2 - CIA increases mortality in APP/PS1 mice**. Survival analysis of wild-type (Tg-) and APP/PS1 (Tg+) mice treated with vehicle or CIA at 2 months of age (Tg-vehicle, *n *= 30; Tg-CIA, *n *= 34; Tg+ vehicle, *n *= 30; and Tg+ CIA, *n *= 34). Click here for file
