# Supplementary material for: Effects of collagen-induced rheumatoid arthritis on amyloidosis and microvascular pathology in APP/PS1 mice
Source: BMC Neurosci. 2011 Oct 27;12:106. doi: 10.1186/1471-2202-12-106 (PMC3217907; doi:10.1186/1471-2202-12-106)
Supplement: Additional file 1 — Figure S1 - Transient increase of microglial cell proliferation in wild-type mice treated with CIA. Photomicrographs of the dentate gyrus molecular layer immunolabeled with Mac-1 after second injection of CII in CFA in 2-month-old wild-type mice. The number of Mac-1-immunoreactive microglial cells was increased within 1 d after second injection of CII in CFA. This increase remained for over 1 wk, but disappeared within 2 wks (n = 4 per group). Scale bar = 200 μm. [file 1471-2202-12-106-S1.PDF]

Figure S1

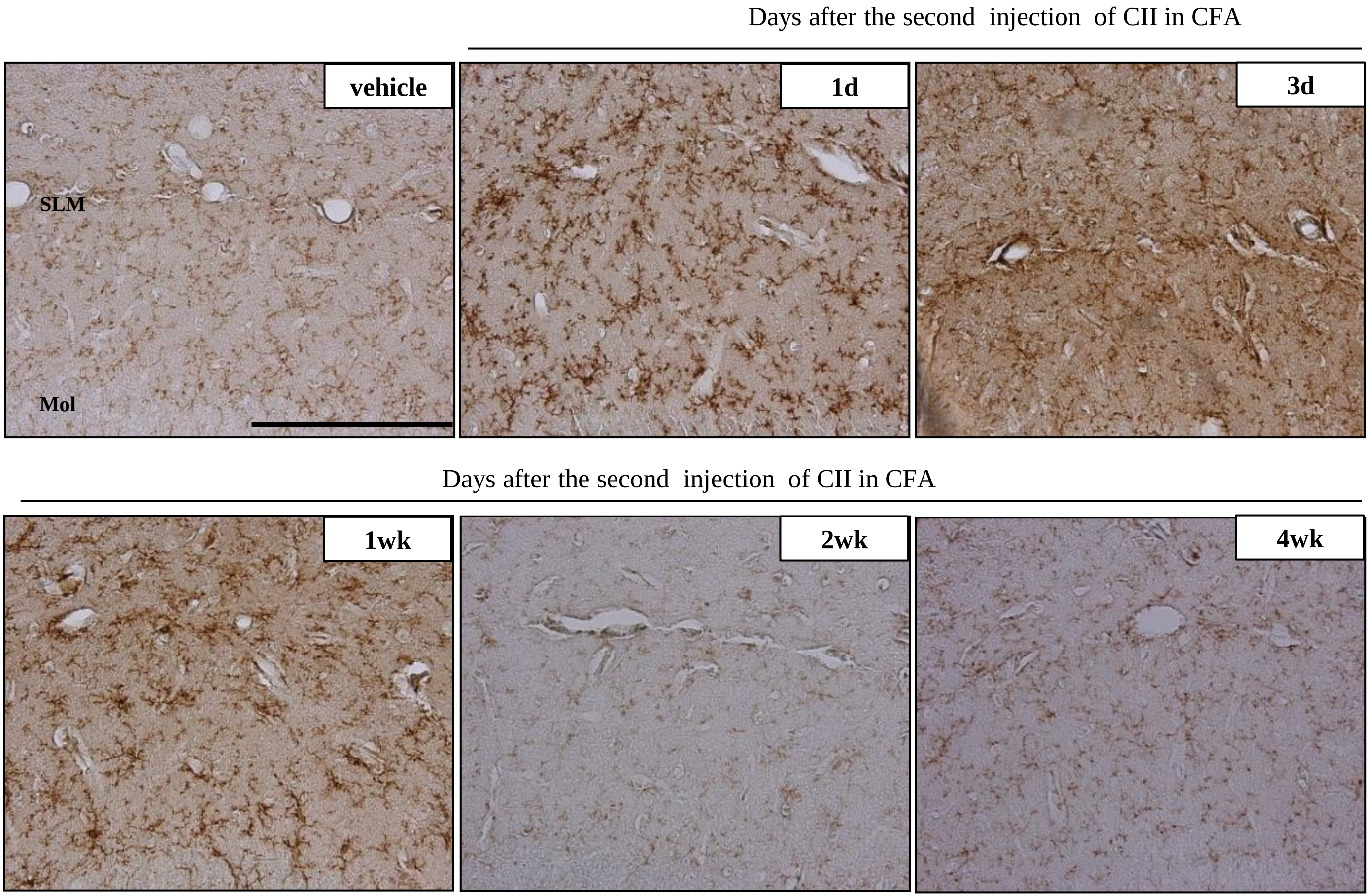

Days after the second injection of CII in CFA
vehicle
1d
3d
SLM
Mol
Days after the second injection of CII in CFA
1wk
2wk
4wk
