# Supplementary figures and images for: Effects of collagen-induced rheumatoid arthritis on amyloidosis and microvascular pathology in APP/PS1 mice
Source: BMC Neurosci. 2011 Oct 27;12:106. doi: 10.1186/1471-2202-12-106 (PMC3217907; doi:10.1186/1471-2202-12-106)

**Figure S2**

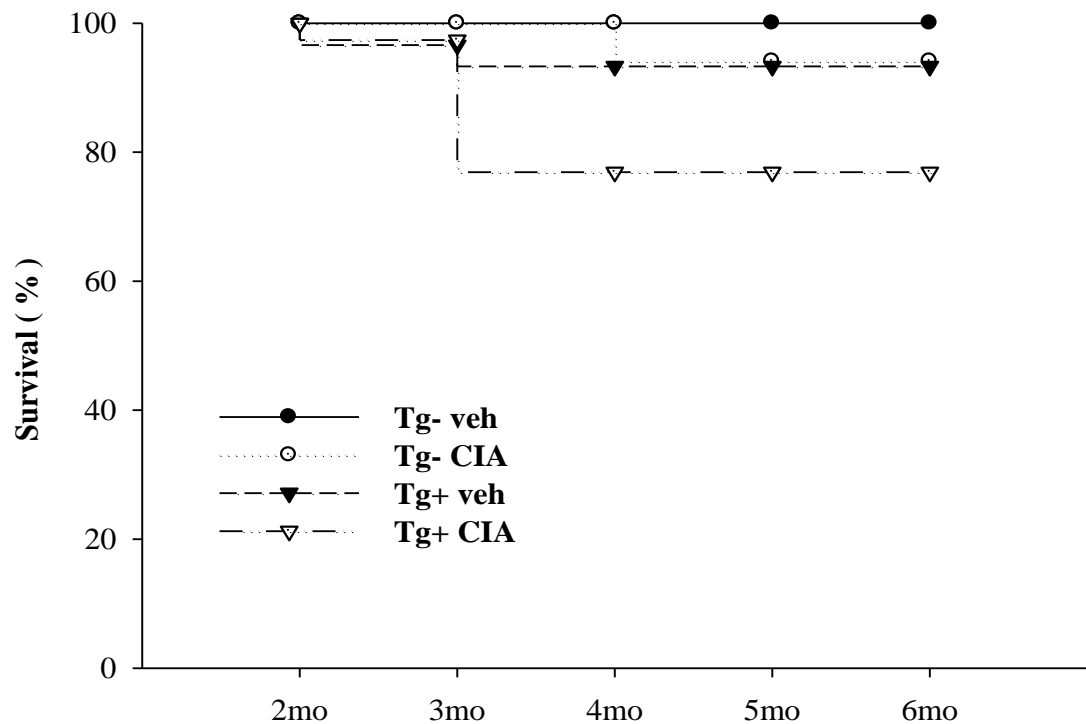

Supplement: Additional file 2 — Figure S2 - CIA increases mortality in APP/PS1 mice. Survival analysis of wild-type (Tg-) and APP/PS1 (Tg+) mice treated with vehicle or CIA at 2 months of age (Tg-vehicle, n = 30; Tg-CIA, n = 34; Tg+ vehicle, n = 30; and Tg+ CIA, n = 34). [file 1471-2202-12-106-S2.PDF]
